# The Cost and Cost-Effectiveness of Vitamin A Supplementation: An Assessment of a Vitamin A Days-Plus Event in Burkina Faso

**DOI:** 10.1177/03795721251355015

**Published:** 2025-08-08

**Authors:** Stephen A. Vosti, Mira Korb, Melissa Baker, Rolf Klemm, Romance Dissieka, David Doledec, Regina Khassanova

**Affiliations:** 1 Department of Agricultural and Resource Economics, 8789 UC Davis, Davis, CA, USA; 2 Institute for Global Nutrition, 8789 UC Davis, Davis, CA, USA; 3 478019 Helen Keller International, Nairobi, Kenya; 4 478019 Helen Keller International, New York, NY, USA; 5 478019 Helen Keller International, Ouagadougou, Burkina Faso

**Keywords:** vitamin A supplementation, costs, coverage, cost-effectiveness, Burkina Faso

## Abstract

**Background:** Campaign-based vitamin A supplementation (VAS) programs are under pressure to reduce costs and increase coverage. **Objectives:** This study examined coverage, costs, cost-effectiveness, and cost burdens of a campaign-based VAS event (JVA+) in the Yako and Kombissiri health districts of central Burkina Faso. **Methods:** Data were collected from groups of JVA+ event implementers and caregivers. Post-event coverage surveys measured coverage; spatially scaled primary data provided estimates of costs. Costs of caregiver participation were measured. Data were provided by all actors involved in the design and implementation of the JVA+ event. **Results:** Overall, 88% of the target age group was covered. Overall coverage did not differ across health districts but was lower in urban areas. Children 6 to 11 months of age had lower coverage, especially in urban areas. The VAS event cost ∼137k USD. Average cost per child reached was 1.34 USD, ranging from 1.19 USD (Yako) to 1.62 USD (Kombissiri). National costs, with international support, covered VA capsules and community health worker salaries. Community stakeholders incurred administrative and transportation/communication costs; regional and district-level stakeholders made small contributions. Caregivers in rural areas contributed significant amounts of time (∼20% of total program costs in some areas). **Conclusions:** The vast majority of Burkinabe children suffer from vitamin A inadequacy. JVA+ events can be generally effective in distributing twice-annual VAS, but are expensive and heavily reliant on international assistance, and are unsustainable. Young children were consistently under-reached, especially in urban areas. Costs to caregivers were high in rural areas. Evidence-based, area-specific changes in program design could increase coverage and efficiency.

## Plain Language Title

High-cost, twice-annual vitamin A supplementation campaigns can fill this micronutrient gap in Burkina Faso, but they are expensive and unsustainable. Ways must be found to reduce costs and increase coverage.

## Plain Language Summary

Vitamin A deficiency remains a significant public health concern globally, causing avoidable child morbidity, underdevelopment, and child deaths. The World Health Organization has recommended twice-annual VAS; the only viable delivery options currently in Burkina Faso are campaign-based VAS events, but costs are high and current programs may be unsustainable. This paper reports on a study that measured the coverage, costs, and cost-effectiveness of a recently implemented VAS delivery event in the Yako and Kombissiri health districts in central Burkina Faso. The program was expensive to design and implement, and the effectiveness and cost-effectiveness varied across districts, across rural-urban areas, and across sub-groups within the targeted child age cohort (6 to 59 months of age). For the overall sample, cost per child reached was 1.34 USD, and ranged from 1.45 USD in rural Kombissiri to 0.93 USD in urban Kombissiri. Options for increasing coverage and improving cost-effectiveness are discussed.

## Introduction

Micronutrient deficiencies, particularly vitamin A deficiency, are significant public health concerns globally, with implications in particular for early-childhood development, morbidity, and mortality,^1 [Bibr bibr2-03795721251355015] [Bibr bibr3-03795721251355015] –4^ and perhaps more broadly.^5^ Dietary inadequacies are present in many low- and middle-income countries (LMICs) and are chiefly responsible for many micronutrient deficiencies.^6,7^ Child health in general is a serious public health concern in Burkina Faso,^8,9^ and micronutrient dietary inadequacy is especially high, e.g., an estimated 91% of young children suffered from vitamin A inadequacy in 2018/2019.^10^ Although various strategies, such as dietary changes, food fortification, and biofortification can help address micronutrient inadequacy,^11^ they all face challenges, especially in LMICs.^12–19^ For instance, improvements in dietary intake patterns can be difficult for income-constrained families, and large-scale food fortification programs may have limited and inequitable reach (e.g., Adams et al.^10^ for Burkina Faso), and industries face compliance issues vis-à-vis established national fortification standards.^20,21^ Biofortification and agronomic biofortification may have potentially large and equitable reach, but producer and consumer adoption issues can limit uptake.^22^ Additionally, the amounts of vitamin A that these programs deliver to children is limited by fortification standards and plant biology (biofortification and agronomic biofortification), by the small portions of foods consumed by young children, and by micronutrient absorption. Hence, even programs with large and equitable reach may not deliver the amounts of micronutrients that are sufficient to achieve dietary adequacy among children broadly.^23^ While these alternative programs, individually or in combinations, have the potential to reduce vitamin A in adequacy in at-risk child populations, aside from the challenges noted above, all of these programs take time to design and implement, in some cases many years, e.g., biofortification programs.^15,24,25^

As policymakers consider various programs to address vitamin A dietary inadequacy and child mortality (in particular), the World Health Organization, with operational guidance from the Global Alliance for Vitamin A, has recommended twice-annual vitamin A supplementation (VAS) through campaign-based delivery platforms.^26–28^ However, the high costs of these programs and their dependence on international funding have led to the phasing out of VAS campaigns in many countries.^29^ To make VAS programs more cost-effective,^a^ organizations are exploring ways to integrate them into existing child healthcare delivery service platforms and identify cost-sharing options among local, national, and international stakeholders^30,31^ but these options, too, face challenges.^32,33^

In this study, we looked *within* an existing campaign-based program to better understand program costs and coverage, and to search for opportunities to increase coverage and to enhance efficiency. More specifically, Burkina Faso has been delivering VAS to children via twice-annual campaigns since 2006. In 2011, the Ministry of Health (MoH) launched twice-annual JVA+ days that delivered an enhanced package of products and services door-to-door to young children and their caregivers—deworming tablets (Albendazole) and VAS were delivered to children, and information (e.g., on child nutrition) was delivered to caregivers—and also screened children for acute malnutrition using middle-upper arm circumference tape measures.^34^ Cadres of community health workers (CHWs) were recruited and paid a monthly wage to deliver JVA+ and other products and services in rural areas; in urban areas community distributors (CDs) were paid a daily wage to do the same. In rural areas the campaigns lasted for approximately 30 days, while in urban areas the campaigns were undertaken in approximately 4 days.^34^ According to national data, VAS provided during JVA+ events reach all or nearly all children. However, post-event coverage surveys (PECS) reported lower levels of coverage nationally for all children in the 6 to 59-month age group, and substantial variation across age sub-groups, and among urban and rural inhabitants. For example, according to PECS data collected after the first round of the 2018 JVA+ event, 70% of all children in the target age group were covered, but only ∼37% of children 6 to 11 months of age were covered in urban areas, while ∼78% of children 12 to 59 months of age were covered in rural areas.^34^ These differences in coverage for a given JVA+ event raise questions and perhaps opportunities. For example, were there efficiency differences in JVA+ programs as they played out in rural versus urban areas? If so, what might have contributed to these differences, and might these contributing factors offer suggestions for improving future JVA+ events?

This paper explores these and related issues in the context of JVA+ event that took place from December 2021 to January 2022, with particular focus on 2 health districts, and used these results to identify options for reducing and possibly shifting the burden of JVA+ costs while also increasing VAS coverage.^b^

## Methods

### Overview

To estimate the costs and cost-effectiveness of the delivery of VAS^c^ in the context of a specific JVA+ event, 3 types of data were needed: VAS coverage, the budgetary costs of the JVA+ event (regardless of who paid them), and the opportunity costs of unpaid participants in VAS distribution and the unpaid caregivers of VAS recipients. The study focused on 2 health districts, Yako and Kombissiri, out of the 70 districts in Burkina Faso where the events were held. These districts were chosen based on security and accessibility for data collection teams, and also to leverage existing collaborations between key health sector actors, supporting organizations, and the health district authorities.^d^

**Figure 1.**
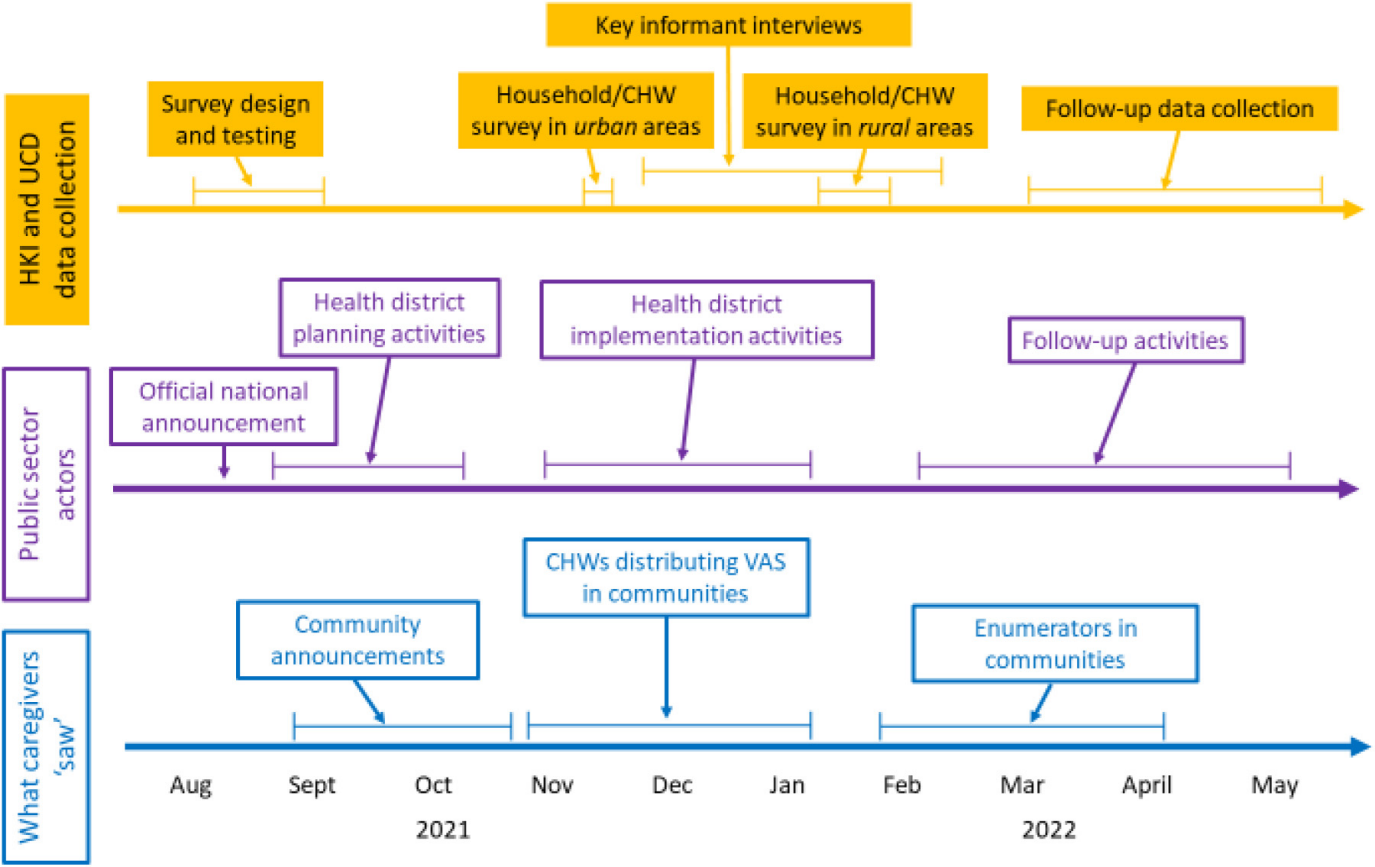
Temporal flows of information and research activities, by group of actors.

The project was comprised of several overlapping ‘layers’ of activities and occasionally overlapping sets of actors. Figure 1 (top row, in yellow) shows the research planning and data collection activities associated with the JVA+ event. Planning began even before the official announcement of the JVA+ event. Then, to collect program activity and cost data, surveys were designed, tested, and administered to CHWs in rural areas and to CDs in urban areas, and semi-structured key informant interviews were conducted among primary actors involved in the JVA+ event. Finally, PECS were conducted shortly after the event to gather information on whether targeted children received VAS (coverage) and the opportunity costs of caregivers. Public-sector activities associated with the JVA+ event are presented in the middle row of Figure 1 (in purple). The final row (in blue) reports what the caregivers and other participants witnessed during the event, including VAS distribution and other products/services provided by CHWs, and the collection of PECS and other data by field enumerators.

### Sampling

**Figure 2.**
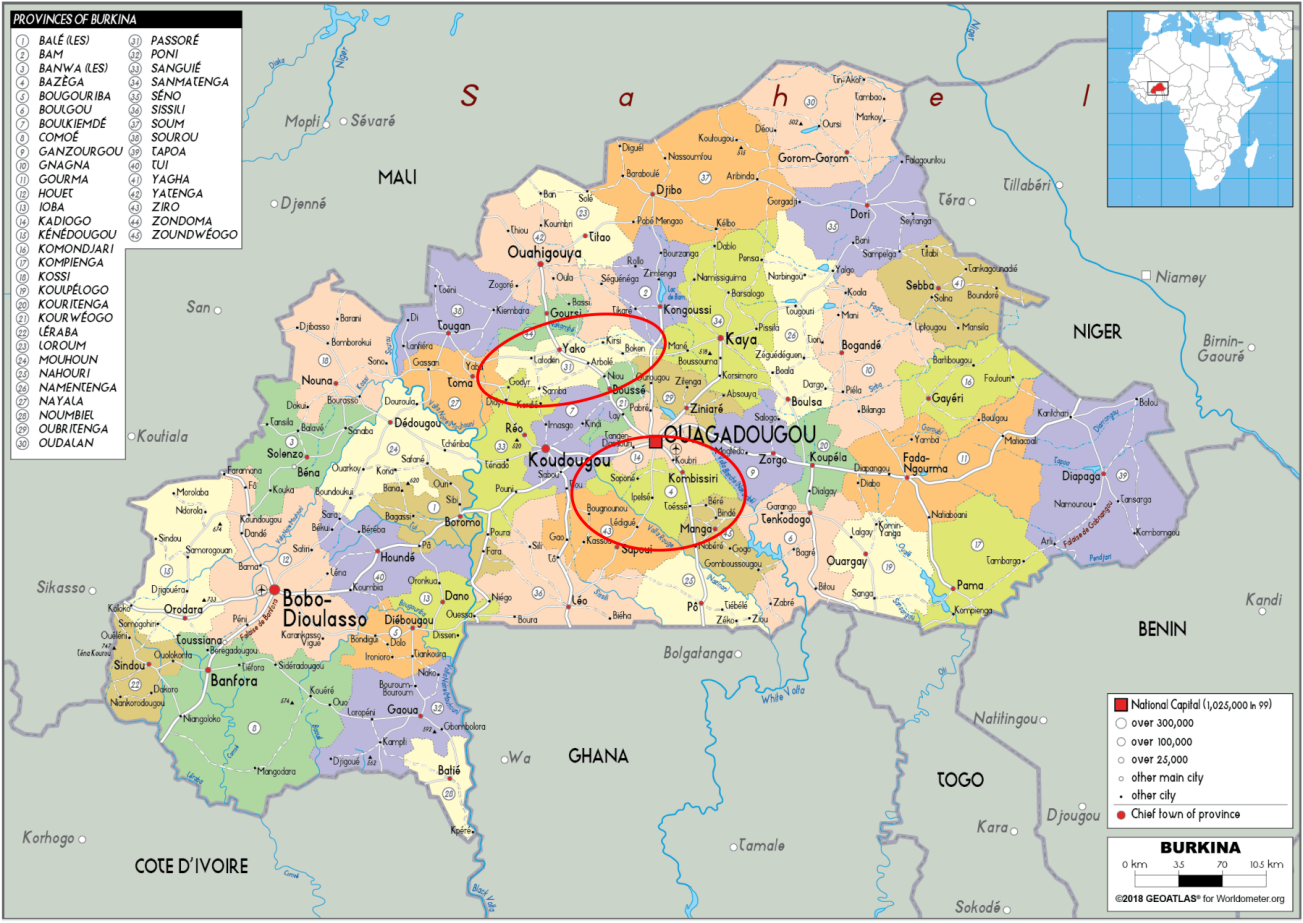
Map of Burkina Faso, with Yako and Kombissiri districts identified.

The PECS used a two-stage, stratified cluster random sampling methodology to select sampled households within selected geographic areas, following the World Health Organization protocol for vaccination coverage.^35^ The primary sampling units (PSUs) were enumeration areas, as defined by the Burkina Faso National Institute of Statistics and Demography, and households were the secondary sampling units. The sample was stratified by the 2 purposively selected health districts, Yako and Kombissiri (as shown in Figure 2), and a probability-proportional-to-size method was used to ensure equal chances of sampling for each enumeration area based on its population size. The sample was also implicitly stratified by rural and urban areas to ensure that the ratio of urban-to-rural respondents in the survey matched the ratio of urban-to-rural respondents in each stratum.^e^

We implemented the two-stage cluster sampling method, first by randomly selecting 77 enumeration areas from each of the 2 health districts, Yako and Kombissiri, which were classified as either rural or urban areas.^36^ Then, enumerators conducted censuses to identify eligible households in each selected enumeration area. Eligibility was based on whether the household had at least 1 child between the ages of 6 and 59 months.^f^ Finally, we randomly selected 11 households from the set of eligible households in each enumeration area. In total, the survey included 1696 households across 2 health districts: 845 in Yako and 851 in Kombissiri. Of these 845 households in Yako, 219 were in urban areas and 626 were in rural areas. Of the 851 households in Kombissiri, 222 were in urban areas and 629 were in rural areas.^g^

For the CHW survey, once enumeration areas were chosen using the procedure outlined above, rural health clinics (CSPS) that served the chosen enumeration areas were selected to be sampled. From each CSPS, 1 to 2 CHWs were randomly selected from a complete list of CHWs eligible to be surveyed. For key informant interviews, purposive sampling techniques identified individuals with direct involvement in the JVA+ event. To minimize recall bias, all data collection activities began at the end of January 2022, immediately following the conclusion of JVA+ event.

### VAS Coverage

In this study, VAS coverage refers to the proportion of children 6 to 59 months of age who received VAS during the JVA+ event conducted from December 2021 to January 2022, out of all targeted children 6 to 59 months of age in the Yako and Kombissiri health districts. Structured household surveys were conducted among a representative sample of caregivers of the targeted children. The enumerators showed samples (or pictures) of both dosages of vitamin A capsules (100 000 and 200 000 International Units, with the former administered to children 6 to 11 months of age) to the caregivers and asked if each child recorded in the household roster had received either type of capsule during the most recent JVA+ event, regardless of *where* the child received it.^h^

### VAS Program Costs

Costs associated with the JVA+ event were incurred at the national, regional, district, and local levels. We collected data on all costs related to the planning and management of the JVA+ event, and the final administration of VAS using (a) structured surveys administered to caregivers, CDs in urban areas, and CHW in rural areas, and (b) structured key informant interviews with health facility staff at CSPSs, health management teams at the health district and regional levels, as well as staff from Helen Keller International and UNICEF who helped plan, partially financed, and oversaw JVA+ activities at the national level.

The caregiver surveys collected information on the time that caregivers spent waiting for their child/children to receive VAS. This included the time spent waiting for the CHW to arrive at their house, and the time spent waiting for the CHW to complete the VAS administration once they had arrived. Fewer than 1% of caregivers reported taking their child to receive VAS at a location away from the homes (e.g., school, neighbor's house), however, to account for these additional costs, the surveys collected data on time spent and transportation costs associated with traveling to these locations. Since most caregivers did not report earning a wage, to value caregivers’ time spent on VAS-related activities, essentially the opportunity cost of their time, we used the average wage earned by individuals with no formal education, as reported in the Enquête Régionale Intégrée sur l’Emploi et le Secteur Informel.^37^^,^^i^

The CD/CHW surveys collected data on the time dedicated to, and on the out-of-pocket cash spent during, the JVA+ event. To estimate the financial costs borne by CDs/CHWs during JVA+, we multiplied the average expenditure on transportation and communication per health worker by the total number of health workers for each health district, separately for rural and urban areas. Expenditures on transportation were calculated as the sum of transportation costs paid by CDs/CWHs to travel to households to administer VAS, and to health facilities for JVA+ -related activities. Transportation costs associated with attending planning and organizational meetings were reimbursed, and therefore were excluded from this sum. Expenditures on communication were given by the total cost of cell phone airtime for communications relating to JVA+, as reported by CDs/CHWs. We also collected data on the total number of working days dedicated by CDs/CHWs to the JVA+ event. CWHs working in rural areas were paid 20 000 CFA for the entire 4-week period VAS distribution; CDs working in urban areas were paid 3000 CFA per day for the approximately four-day period of urban VAS distribution. Based on these payment rates, we estimated the daily salaries of CDs/CHWs. Thus, for the purposes of cost accounting for CDs/CHWs, there are 2 relevant types of costs: (1) financial costs borne by CDs/CHWs through their *own* expenditures on transportation and communication during JVA+ and (2) salaries paid to CDs/CHWs for their involvement in the JVA+ event. All of these costs were included in estimating the total cost of the JVA+ event.

To collect data on the cost of supplies used to distribute VAS during JVA+, structured key informant interviews were conducted among staff from Helen Keller International and UNICEF, as well as regional and health district nutrition coordinators. Supplies included vitamin A capsules (100 000 IU and 200 000 IU), T-shirts, banners, masks, scissors, pens, posters, soap, and alcohol cleaning gel. In addition to data on the unit costs and the number of units purchased or acquired, we also collected information on the cost of storing and transporting supplies.

Data on other operational costs associated with the JVA+ event were collected via structured key informant interviews with staff from Helen Keller International, and regional and health district nutrition officers, as well as nutritionists, head nurses, or administrators at each health facility included in the study. In these surveys, respondents were asked to identify all administrative personnel in their respective offices or facilities who were involved in the planning, management, and implementation of the JVA+ event that took place from December 2021-January 2022. We requested lists of job titles,^j^ the number of personnel in each job, days spent working on JVA+, and the average hours per day spent working on JVA+. Information was also collected on overhead costs per month, including rent, warehouse payments, utilities, maintenance, water, and office supplies. Finally, these surveys collected data on vehicle insurance, maintenance, and rental rates for 3 vehicle types: Bikes, motorbikes, and cars/trucks. We inquired about the numbers of each type of vehicle, monthly and annual costs of maintenance and insurance, and the days or fractions of days that each vehicle type was used during the JVA+ event.

Finally, key informants were interviewed about the costs of meetings related to the planning and management of the JVA+ event. The interviews focused on 3 main types of activities: meetings, transportation, and social mobilization. First, data collection focused on the costs associated with hosting and attending meetings or trainings for the JVA+, including materials, meals, and snacks/tea/coffee. Any per diems or allowances associated with the meetings/trainings were included in the salary and allowance data described above. Second, data were collected on transportation costs associated with trips taken during JVA+ event. These transportation costs covered fuel expenditures, but not the vehicle rental, insurance, or maintenance costs described above. Third, respondents were asked about expenditures on social mobilization activities associated with JVA+, such as paying for posters or banners, TV or radio ads, public announcements via town criers, entertainers, distributing letters or pamphlets, or making phone calls.

### Cost-Effectiveness of the JVA+ Event

Cost-effectiveness was defined as the cost per child who received^k^ VAS during the JVA+ event. Cost-effectiveness was estimated for each health district (Yako and Kombissiri) and for rural and urban areas. More specifically, cost-effectiveness for a given district and area type was calculated as the ratio of the total cost of the JVA+ event in that health district and area type (urban or rural) to the number of children 6 to 59 months of age who received VAS in that health district and area type.

### Coverage

VAS coverage rates (the denominator of the cost-effectiveness measure) by health district and area type were estimated by multiplying the proportion of surveyed children 6 to 59 months of age that received VAS by the total number of children 6 to 59 months of age in each health district and area type. More specifically, let *d* represent the index for the health district (either Kombissiri or Yako) and *a* index the area type (either rural or urban). If we define $\;P_{da}$ as the proportion of surveyed children 6 to 59 months of age that received VAS, and $\;N_{da}$ as the total number of children 6 to 59 months of age in a health district, then the VAS coverage for health district *d* and area *a* is given by $\;Coverage_{da}=\;P_{da}\;\times N_{da}$ .

### Spatial and Temporal Scaling of JVA+ Event Costs

Some JVA+ event costs were incurred and collected at the health district level, but other costs were incurred and collected at the national, regional, or CSPS levels. Therefore, obtaining estimates of the total costs of the JVA+ event by health district and by area type (the numerator of the cost-effectiveness measure) required additional assumptions regarding the spatial allocation of costs. To do so, a set of scaling factors were developed and applied to adjust costs to the appropriate health district and area type spatial units. To estimate health district and area type costs from *national or regional costs*, we scaled costs *down* by the number of children 6 to 59 months of age in a given health district and area, as a fraction of the total number of children 6 to 59 months of age in Burkina Faso (for national costs), or in the relevant region (Nord region for Yako health district, Centre Sud region for Kombissiri health district). To estimate costs for area type from health district-level costs, we scaled costs *down* by the number of children 6 to 59 months of age living in a particular area type (rural or urban) in a given health district as a fraction of the total number of children 6 to 59 months of age living in that health district.

To estimate health district and area type costs from data collected from the *subset* of health facilities, we first calculated the average cost per surveyed health facility, by health district and by area type. We then estimated the costs for health facilities within a given health district that were *not* surveyed by multiplying the average cost per surveyed health facility by the number of *non*-surveyed health facilities, for each health district and area type. Summing the actual costs across surveyed health facilities and the estimated costs across non-surveyed health facilities yielded total costs, by health district and area type.^l^

Finally, there were also temporal differences in the reporting of costs associated with the JVA+ event. Most of the data on costs were based on the number of hours, days, or other time units relevant for specific participants in the JVA+ event. For example, data collected included the number of hours and days CHWs worked on the JVA+ event, the number of days specific vehicles were used for JVA+ activities, and the number of hours that caregivers spent waiting for health workers. We scaled this information to report costs that were appropriate to the JVA+ event timeframe. Data on administrative expenses, such as rent and utilities, were collected on monthly and annual timesteps. In these cases, we made a simplifying assumption that 1 month of administrative expenses, excluding salary and allowance costs of administrative personnel (which were included in other cost categories), were associated with the JVA+ event.

## Results

### VAS Coverage

**Figure 3.**
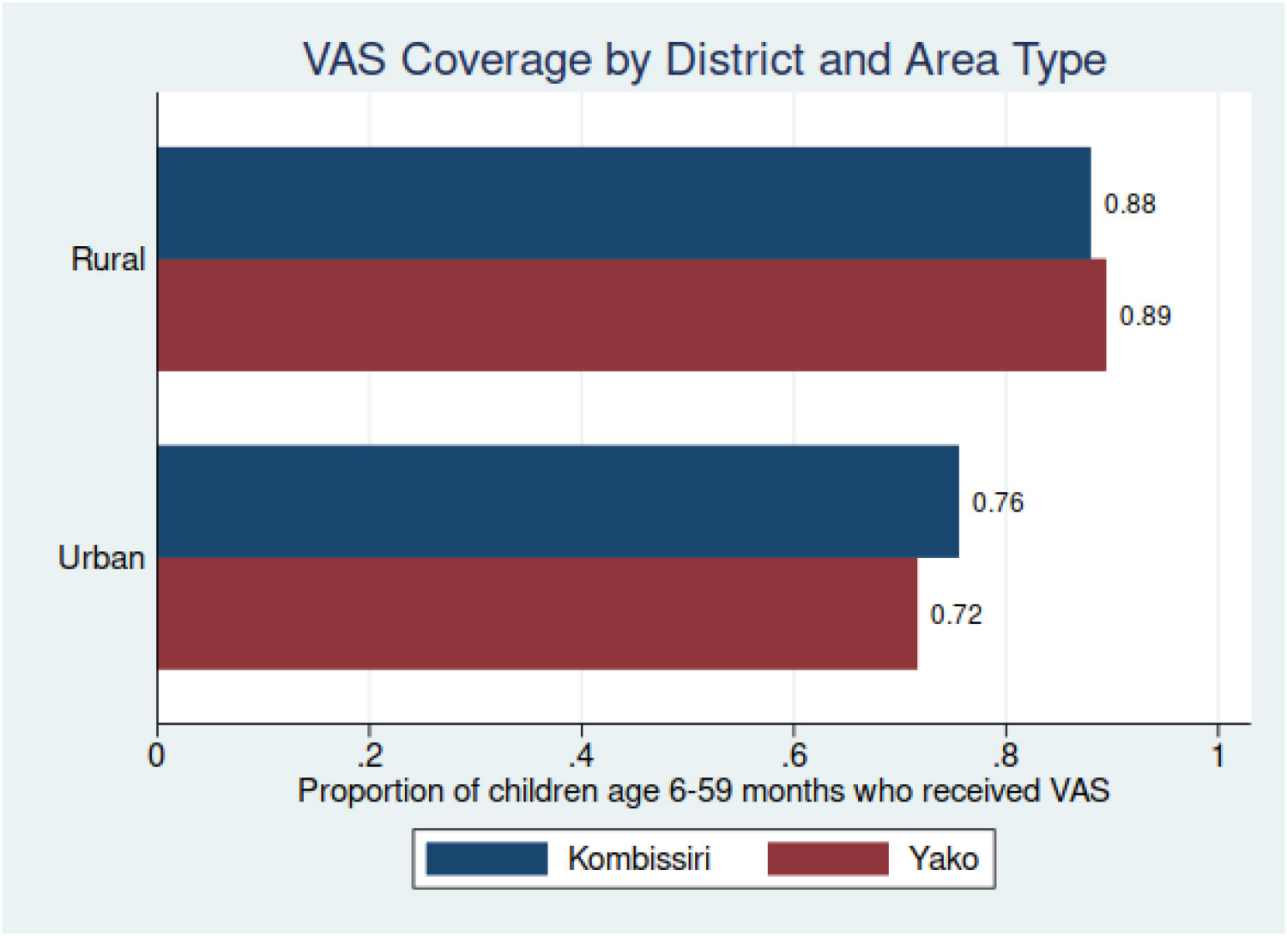
Vitamin A supplementation (VAS) coverage, Kombissiri and Yako health districts, by rural and urban areas.

The vast majority of children 6 to 59 months of age in the health districts of Kombissiri (86.8% [CI = 84.6%, 88.7%] and Yako (88.6% [CI = 86.5%, 90.4%]) received VAS during JVA+ event that took place from December 2021-January 2022.^38^ The total number of children receiving VAS was 102 722, most of whom lived in the Yako health district (68 550, and most of whom resided in rural areas in both districts (95 155). However, these health district-level measures mask heterogeneity in coverage in rural versus urban areas. Overall, coverage was higher in rural areas (89.2% [CI = 87.5%, 90.6%]) than in urban areas (73.5% [CI = 69.5%, 77.1%]) in both health districts. Coverage of rural areas was higher in Yako (89%) than in Kombissiri (88%), whereas coverage of urban areas was lower in Yako (72%) than in Kombissiri (76%) (Figure 3). Nevertheless, coverage was lower in urban areas than in rural areas in both health districts.

**Figure 4.**
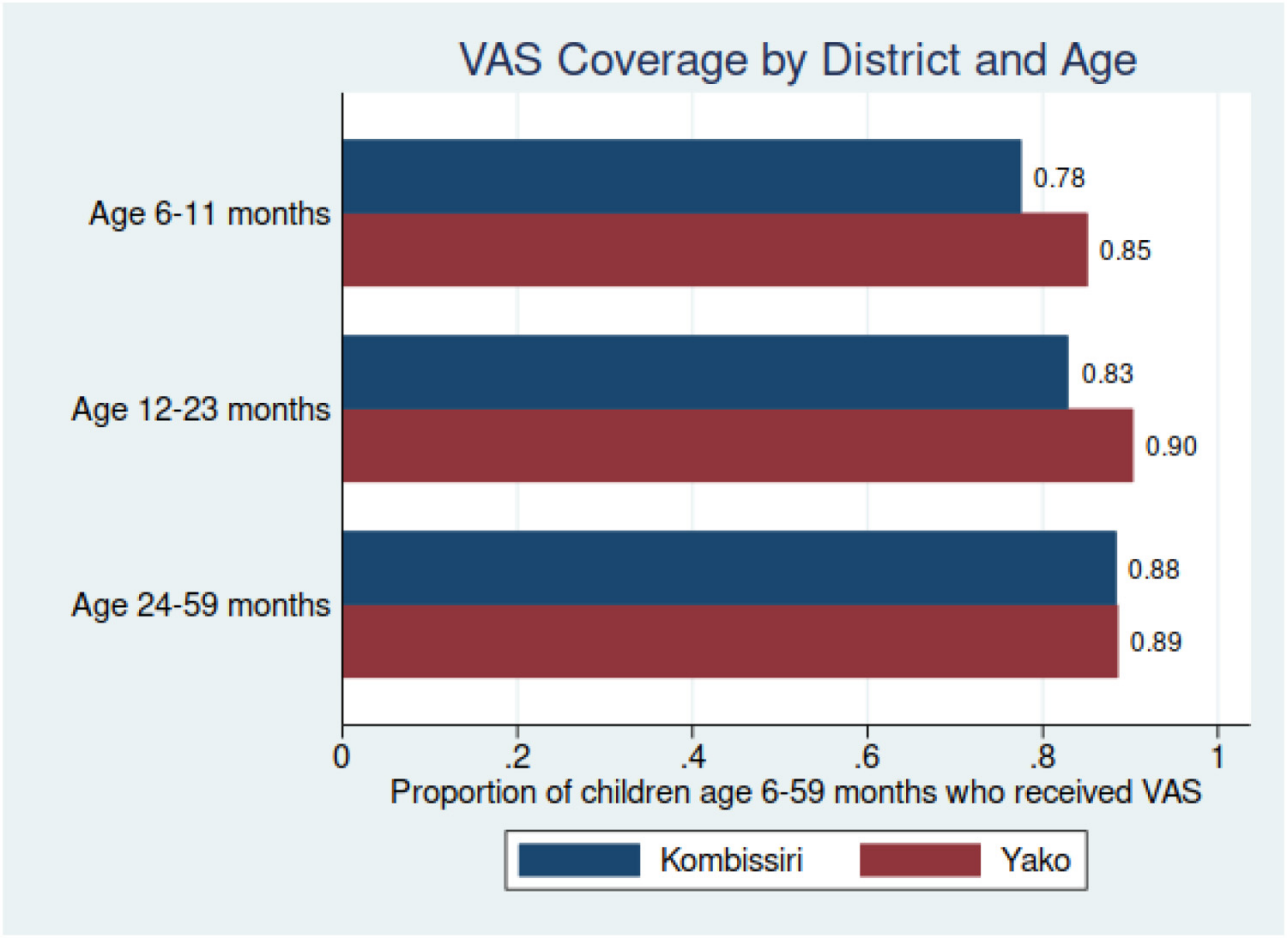
Vitamin A supplementation (VAS) coverage, Kombissiri and Yako health districts, by child age cohort.

Coverage achieved during the JVA+ event also varied across different age groups within the 6-to-59-month target age range. In both health districts, VAS coverage was lowest for the youngest children 6 to 11 months of age (78%-85%) and highest for the oldest children in the targeted group 24 to 59 months of age (88%-90%) (Figure 4). Although there were some differences in coverage between the 2 health districts across all age groups, these differences tended to be small.

**Figure 5.**
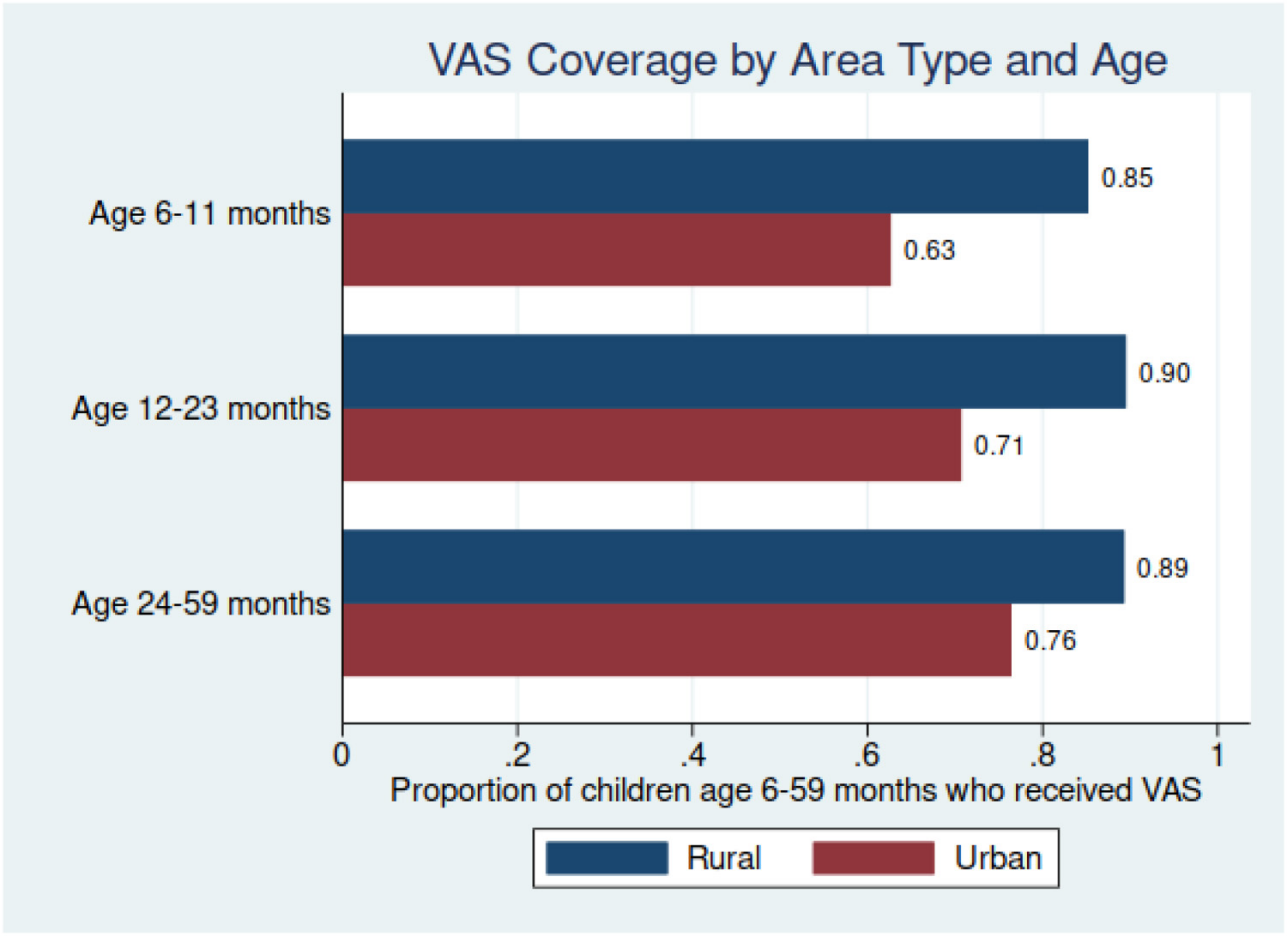
Vitamin A supplementation (VAS) coverage, by age cohort, and by urban and rural area.

When comparing coverage between rural and urban areas (Figure 5), coverage was substantially lower in urban areas for all age groups. This difference in coverage between rural and urban areas ranges from a 12-percentage point difference for children 24 to 59 months of age to a 22-percentage point difference for children 6 to 59 months of age.

### JVA+ Event Costs and Cost Drivers

**Figure 6.**
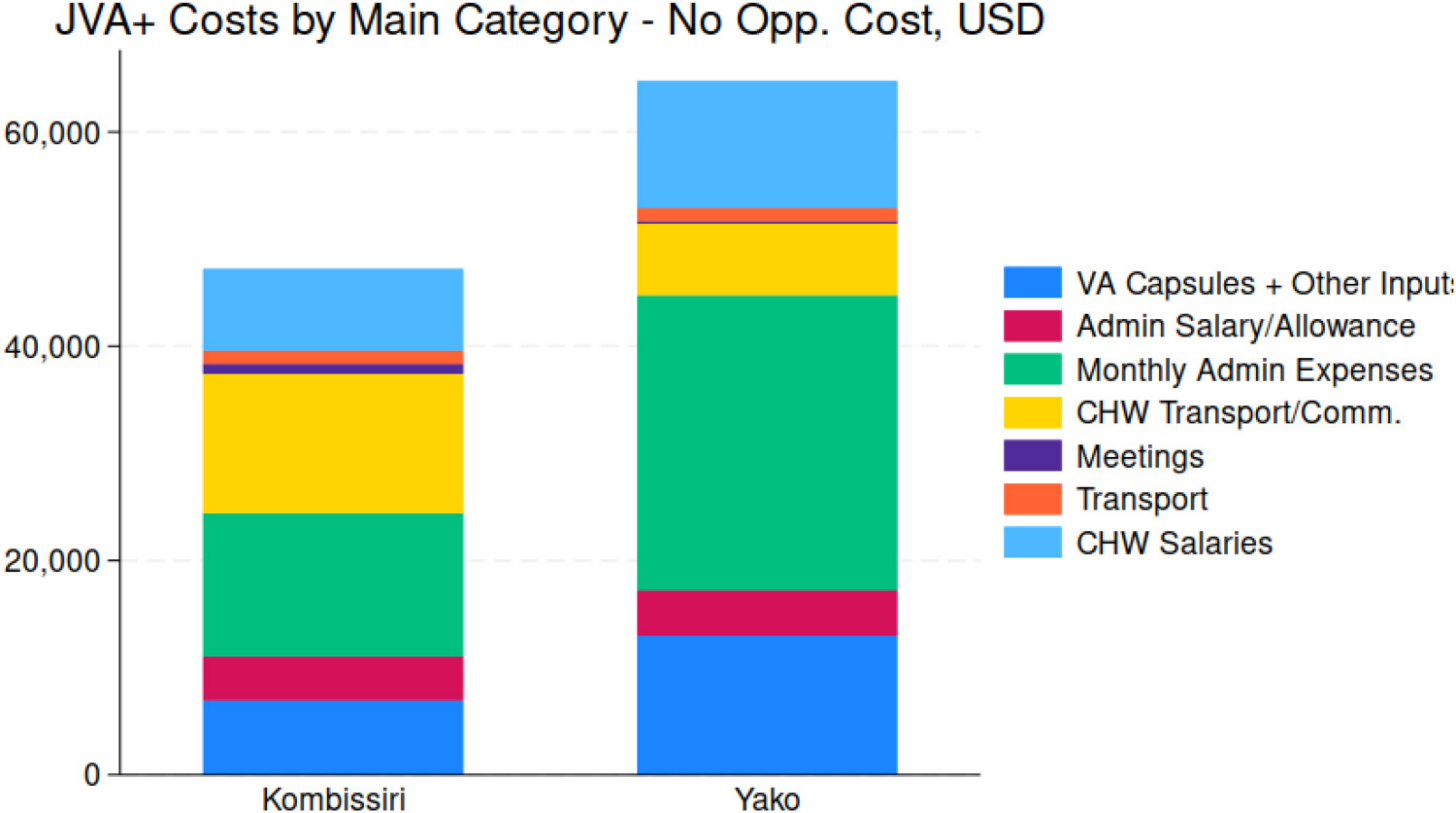
Total JVA+ Program costs, by main cost categories, by health district.

The total cost of the JVA+ event, net of the opportunity cost of caregivers’ time, was 64 919 USD for Yako and 47 598 USD for Kombissiri.^m^ The composition of costs associated with the JVA+ event varied across different locations and activities. In our data collection and analysis, we grouped JVA+ expenditures into several cost categories:^n^ VAS capsules and other supplies, administrative expenses (such as rent, utilities, vehicles), salaries and allowances of administrative personnel, financial costs borne by health workers (such as transportation/communication), meeting and training costs, social mobilization costs, personnel transportation costs (including fuel), and salaries of CDs/CHWs. Detailed cost estimates are provided in Table 1, and Figure 6 presents the total costs and cost composition for each health district. The composition of costs also differed between the 2 health districts, with higher monthly administrative expenses and vitamin A capsule costs in Yako, and higher CHW transportation and communication costs in Kombissiri. Monthly administrative expenses accounted for approximately 20% to 40% of JVA+ costs in both districts, including expenditures on rent, storage, bills, building maintenance, office expenses and other administrative expenses including vehicle rental, maintenance and insurance for vehicles used during the JVA+ event. Higher vehicle-related expenses were a key driver of the total monthly administrative expenses, particularly vehicle rental costs, which accounted for a larger share of vehicle-related JVA+ expenses, compared to vehicle insurance and maintenance costs. Similar patterns emerged for bicycles and cars, but motorbikes were the most prominent contributors to costs, as they were used more frequently and for longer periods than other vehicle types.

**Table 1.** JVA+ Event Costs (USD)—Overall, by Health District, by Urban/Rural Areas.

	Total	
Total JVA+ Costs	137 140	
VA capsules and other input costs	19 974	
Admin salary and allowance	8 326	
Monthly admin expenses	40 915	
Meetings	1 196	
Transport	2 482	
Community health worker (CHW) salaries	19 440	
CHW transport/communication	19 628	
Caregiver opportunity costs	24 623	
Social mobilization	558	
	Kombissiri	Yako
Total JVA+ Costs	55 430	81 710
VA capsules and other input costs	6 977	12 997
Admin salary and allowance	4 077	4 249
Monthly admin expenses	13 390	27 525
Meetings	946	249
Transport	1 264	1 218
CHW salaries	7 596	11 844
CHW transport/communication	12 968	6 660
Caregiver opportunity costs	7 832	16 791
Social mobilization	381	177
	Rural	Urban
Total JVA+ Costs	126 227	10 913
VA capsules and other input costs	17 727	2 247
Admin salary and allowance	7 534	793
Monthly admin expenses	37 654	3 261
Meetings	1 073	122
Transport	1 750	732
CHW salaries	17 820	1 620
CHW transport/communication	19 171	457
Caregiver opportunity costs	23 055	1 569
Social mobilization	450	108

Authors calculations. All costs are reported in 2022 USD; exchange rate 1 XOF = 0.0015 USD.

**Figure 7.**
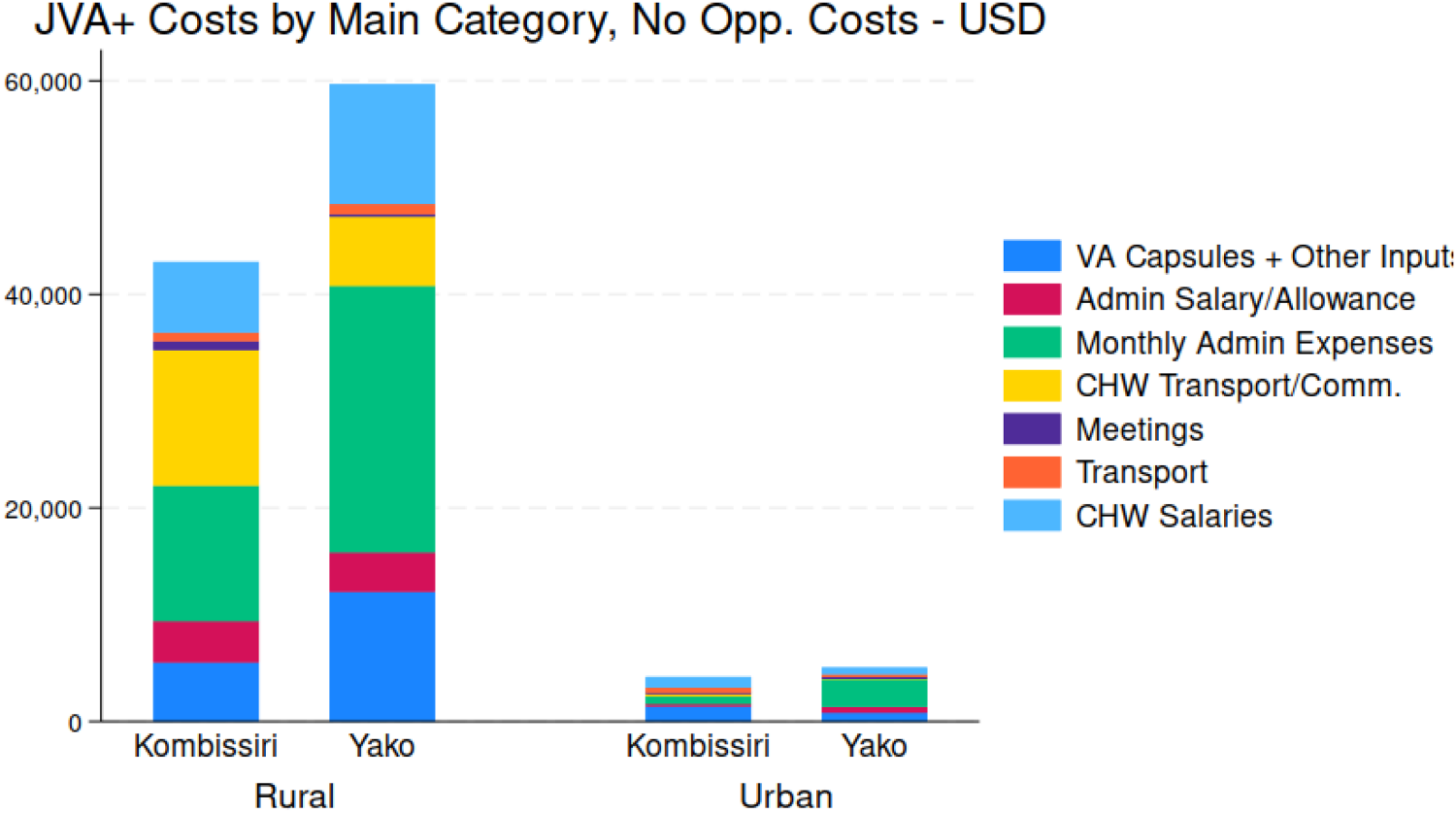
Total JVA+ Program costs, by main cost categories, by health district, and by rural and urban area.

Total costs, again net of caregivers’ costs, were substantially higher in rural areas (103 172 USD) than in urban areas (9344 USD); this difference was mainly attributable to the location of the target population of children. When examining costs by rural and urban areas within districts (Figure 7), the cost composition became more diverse. Total costs across all categories were higher in rural areas than in urban areas, mainly due to the larger populations served in rural areas. In rural areas, monthly administrative expenses comprised the largest share of costs (∼20%-35%). However, in urban areas, monthly administrative expenses, while relatively small compared to those in rural areas, accounted for the largest share of costs in Yako (40%) but only a minimal share in Kombissiri (10%). Across both health districts and area types, supply costs represented a significant share of the total costs, ranging from approximately 15% in urban Yako to 30% in urban Kombissiri. These supply costs were driven by the costs of purchasing vitamin A capsules, which was directly linked to the size of the target child population. In all cases, except for rural Kombissiri, the financial costs borne by health workers (out-of-pocket expenses for transportation and cell phone communication) represented a relatively small share of total costs (5%-10%). However, in rural Kombissiri, this share exceeded 20%. CD/CHW salaries accounted for a similar share across all health district areas, ranging from 15%-20%. Interestingly, while transportation costs seemed almost negligible in rural areas, their share in urban areas was larger (5%-10%).

**Figure 8.**
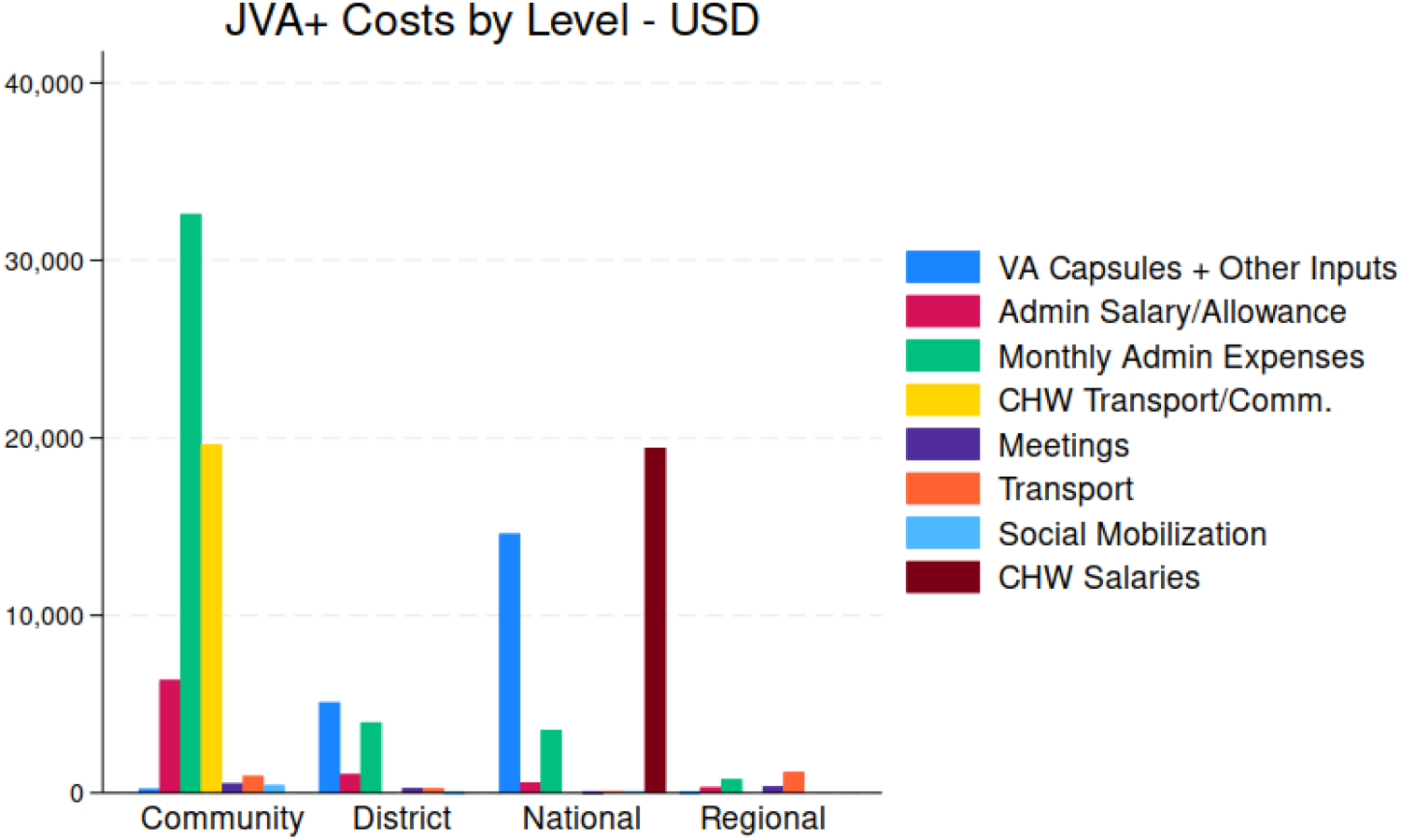
Total JVA+ Program costs, by geographic areas and associated stakeholder groups.

**Figure 9.**
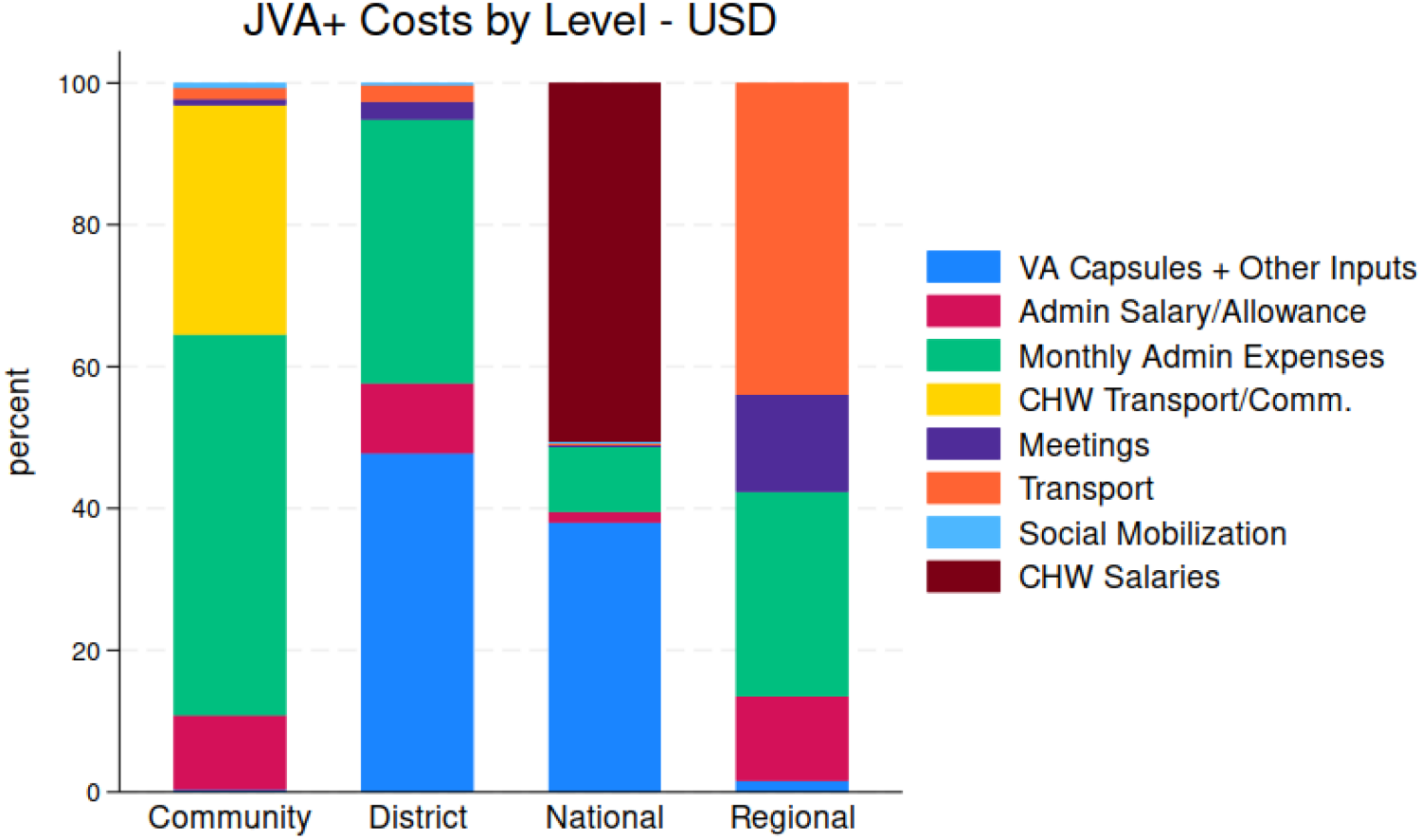
Percent of total JVA+ Program costs, by geographic areas and associated stakeholder groups.

When discussing policies related to the JVA+ event, the issue of the allocation of costs across stakeholder groups often arose. The costs were distributed across different levels, from the community to the national level, with various stakeholders being responsible for different expenses. Figure 8 provides an overview of the spatial distribution of JVA+ event costs, categorized by cost type. The results reveal 2 important findings. First, national and community stakeholders bore the majority of total JVA+ event costs. Costs incurred by district- and regional-level stakeholders were relatively modest. Second, as shown more clearly in Figure 9, the composition of cost covered by different stakeholders varied significantly. For instance, as expected, national stakeholders (with support from international agencies) were primarily responsible for vitamin A capsule costs. On the other hand, communities were responsible for most of the monthly administrative expenses and CHW transportation/communication costs. The salaries of CHW, however, were the responsibility of national stakeholders (in the case of this JVA+ event, with support from the international community).

**Figure 10.**
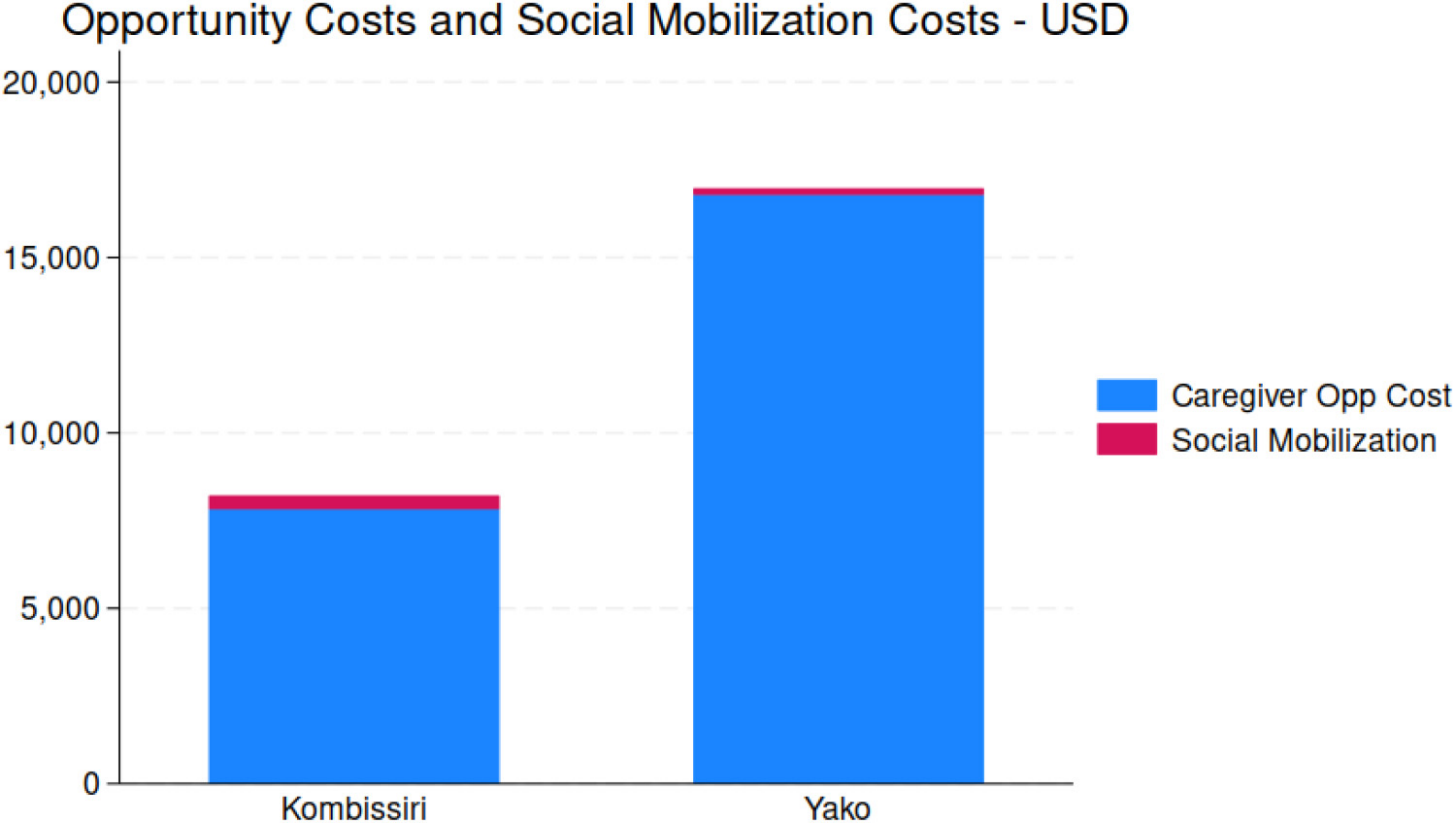
Total caregiver opportunity costs and social mobilization costs, by health district.

**Figure 11.**
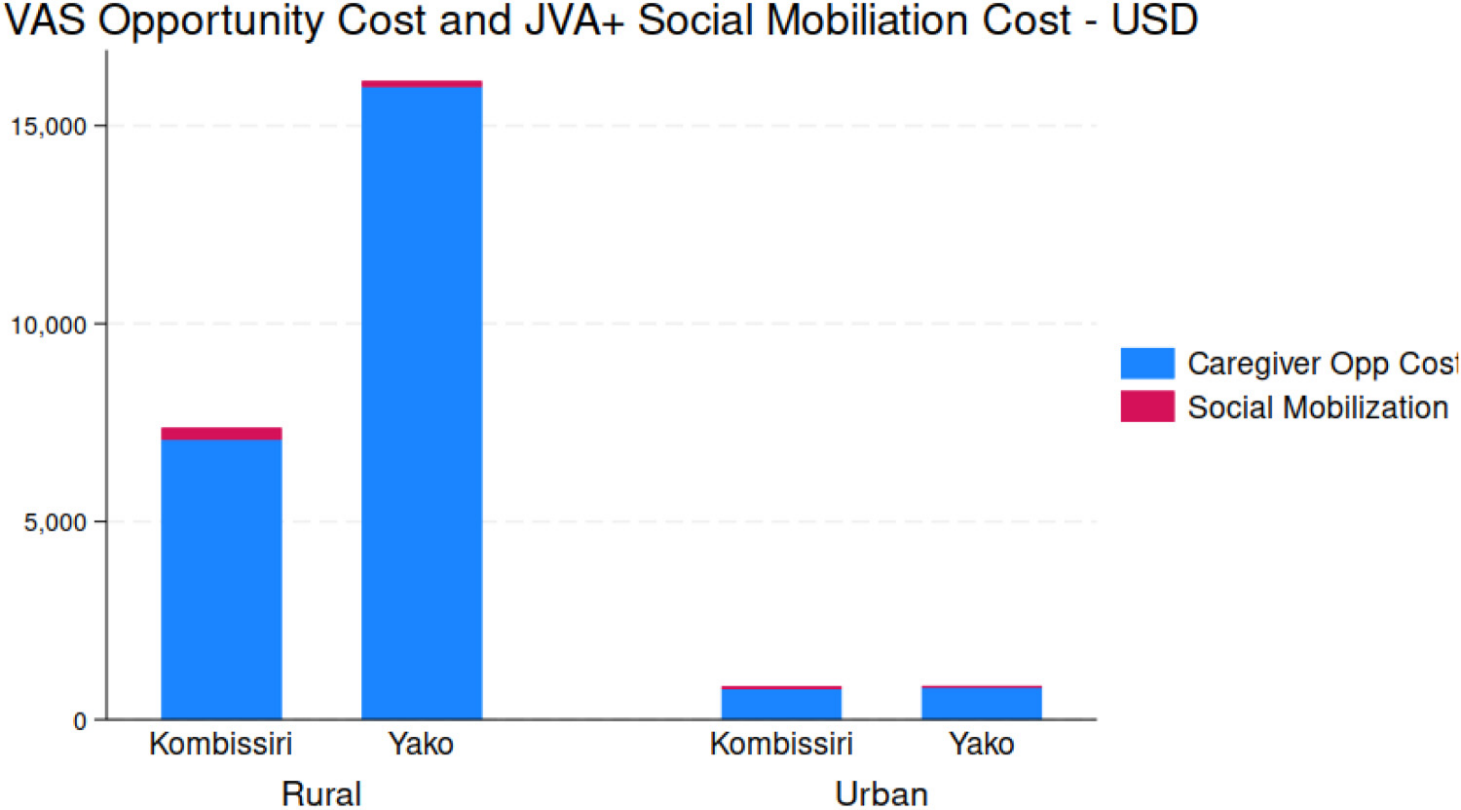
Total caregiver opportunity costs and social mobilization costs, by health district, by rural and urban areas.

In addition to stakeholders previously discussed, caregivers of the children who received vitamin A capsules during the JVA+ events and individuals who participated in social mobilization events also incurred costs.^o^ These stakeholder groups are often forgotten in program and policy discussions. The study collected data on the waiting and travel times of caregivers who left their homes to receive VAS, and the value of their time was estimated based on the wage paid to workers without any formal education. Figure 10 provides an overview of the total opportunity costs borne by caregivers and total social mobilization costs for each health district. Given the larger targeted populations in rural areas, caregivers in rural areas faced higher total opportunity costs compared to those of urban areas. While Yako had higher caregiver opportunity costs, caregivers in both health districts had substantial costs, representing 15% of total JVA+ costs in Yako and 25%; Figure 11 provides spatial snapshots of the cost burden faced by caregivers. In contrast, social mobilization costs were relatively small in both health districts.

Although most caregivers in all locations had short waiting times for CHWs to administer VAS, a significant number of caregivers in rural areas had to wait for several hours, and in some cases even longer for the arrival of CHWs. This was particularly prevalent in rural Yako. See Figure SM6 in the Online Supplemental Material for details.

### Cost-Effectiveness of JVA+ Event

**Figure 12.**
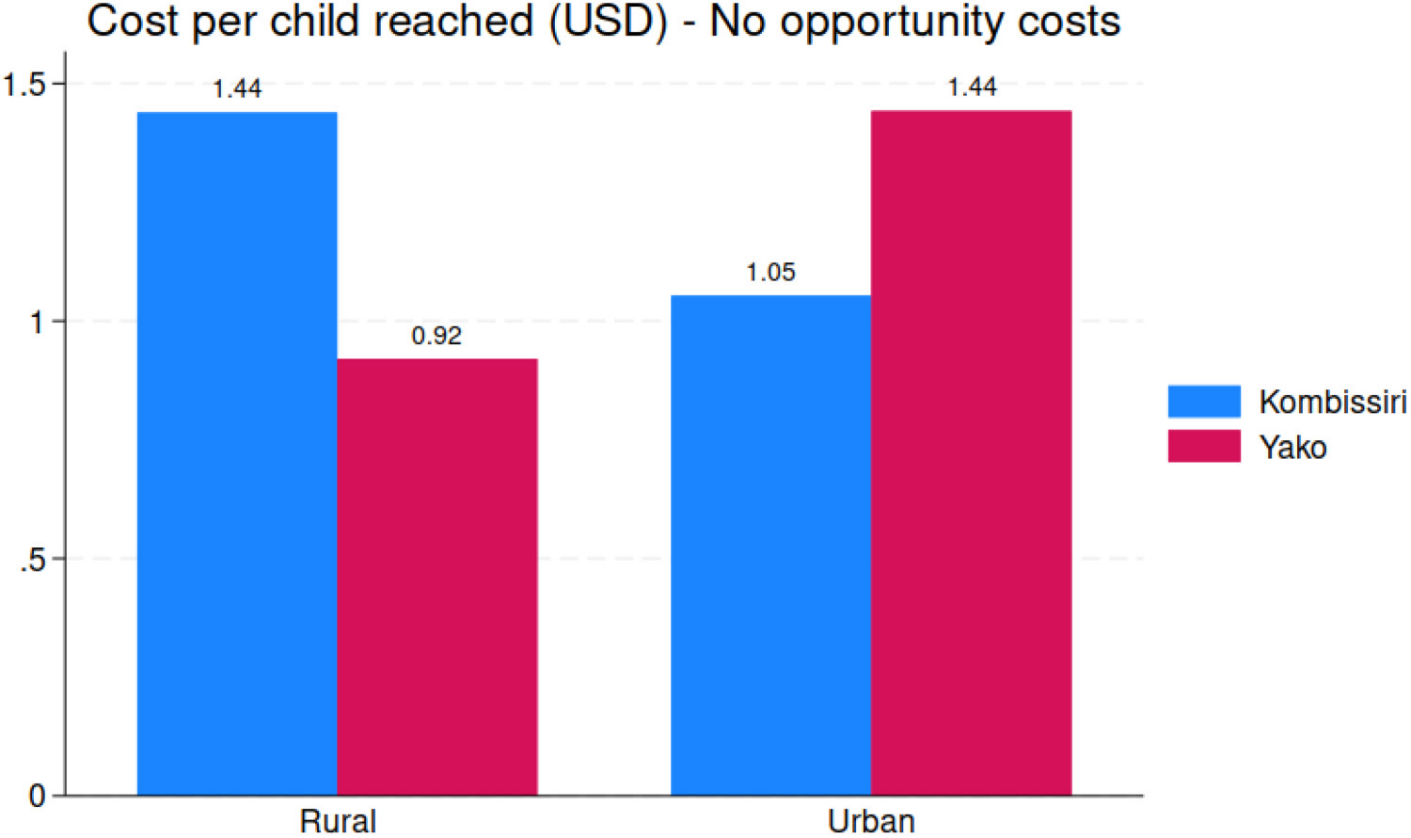
Cost per child reached, rural and urban areas, by health district.

Lastly, we present the results of cost-effectiveness analysis of the JVA+ event, by district, and by rural and urban areas. Our measures of effect are the number of children targeted (the total population of children 6 to 59 months of age) or reached (the number of children who received the age-appropriate dose of vitamin A provided by the supplementation capsules). These serve as the denominators of our cost-effectiveness ratios. The numerators are the total costs of the JVA+ event (in this instance, excluding the opportunity costs of caregivers’ time). The average cost per child *targeted* ranged from 1.05 USD in Yako to 1.40 USD in Kombissiri. The cost per child *reached* ranged from 1.19 USD in Yako and 1.62 USD in Kombissiri. When viewed from an urban versus rural area perspective and reporting cost per child reached, Figure 12 reveals that the higher cost-per-child-reached in Kombissiri is primarily driven by the cost of reaching children in rural areas (1.44 USD vs 1.05 USD). In contrast, the opposite is true for Yako, where it was more costly to reach urban children than those residing in rural areas (1.44 USD vs 0.92 USD).

## Discussion

Vitamin A inadequacy and vitamin A deficiency remain public health problems, although we have less evidence to support the latter than the former .^2,7,39^ The WHO continues to recommend twice-annual doses of VAS for all at-risk children 6 to 59 months of age, setting a target of 80% coverage. In most LMIC settings, to date, the only way to meet this target level of coverage for all children in this age group is via campaign-based distribution of VAS. However, for some time now, stakeholders have been concerned about the cost and cost-effectiveness of campaign-based VAS programs, especially as the original programmatic underpinning of these programs (polio eradication campaigns) have been eliminated and supplemental international funding available to support VAS campaigns in LMICs has declined.^29,40–42^ There are many ongoing efforts to identify ways of effectively and sustainably introducing VAS programs into primary healthcare programs, but challenges exist, especially for older children who no longer routinely visit clinics for scheduled vaccinations.^30,31,33^

This study examined the cost and cost-effectiveness of a campaign-based JVA+ event in 2 health districts in central Burkina Faso in 2021/2022. We measured the costs associated with the JVA+ event, identified the key cost drivers, and determined the stakeholder groups responsible for paying these costs. We estimated cost-effectiveness by linking program coverage and costs. We identified differences across and within health districts (e.g., rural vs urban areas) and sought to understand why these differences might occur, and then to use this evidence to suggest ways to improve the coverage and cost-effectiveness of future JVA+ programs in Burkina Faso.

Our findings indicate that the JVA+ event covered approximately 88% of children in the targeted age group of 6 to 59 months, which is above the level of WHO's target of 80% VAS coverage. Although coverage did not differ statistically across health districts (∼87% in Kombissiri and ∼89% in Yako), urban coverage was lower than rural coverage in both health districts (75% and 72% in Kombissiri and Yako, respectively). Additionally, coverage among those in the lowest target age group (6 to 11 months of age) was lower than the other age cohorts in both health districts, particularly in urban areas. Therefore, some subgroups *within* the 2 health districts did not meet the WHO target of 80% coverage, mainly in urban areas and among very young children. An assessment of a JVA+ event in Burkina Faso in 2018 found similar asymmetries in coverage by age group and by rural/urban setting.^34^

JVA+ total program costs, including caregiver costs, were highest in Yako (∼82 000 USD) compared to Kombissiri (∼55 000 USD), with the size and location of the target child population being the primary cost driver. The cost composition varied significantly across districts, with Yako spending proportionately more on monthly administrative expenses, and relatively less on CHW transportation and communication expenses. These differences were also observed at the rural level. Overall, cost per child reached overall was 1.34 USD, but ranged from 1.45 USD in rural Kombissiri to 0.93 USD in urban Kombissiri, with health-district-level averages of 1.62 USD and 1.19 USD for Kombissiri and Yako, respectively. Extrapolating this to the national coverage of 3.25 million children,^19^ the cost of a single JVA+ event in Burkina Faso would be approximately 4.4m USD, and the annual cost of twice-annual VAS delivered via JVA+ campaigns would be approximately 8.7m USD.^p^

Program costs were allocated differently among stakeholders depending on the location. National costs (with international support) mainly covered vitamin A capsules and CHW salaries. Community-level stakeholders were responsible for monthly administrative expenses and transportation/communications outlays, while regional and district-level stakeholders contributed minimally to the JVA+ event. Caregivers also contributed significantly to the program costs, mostly in terms of their time waiting for CHW to administer vitamin A capsules or traveling to VAS sites. These costs were particularly high in rural areas; indeed, the value of caregiver time devoted to the JVA+ event amounted to approximately 20% of total program costs in one area.

Our findings regarding program costs, cost-effectiveness, the composition of costs, and stakeholder shares of costs are consistent with those of other studies. For example, Neidecker-Gonzales et al.^40^ report a range of cost per VAS capsule delivered from 0.51 USD for Ghana to a high of 2.27 USD for South Africa.^q^ The same study found the high proportion of costs associated personnel and promotional activities, but reported a wide variation across countries regarding the proportion of VAS program costs paid by international stakeholders, e.g., 95% in the case of Guatemala and 10% in the case of the Philippines. Kagin et al.^43^ reported a cost per child reached with 2 rounds of VAS of 1.09 USD in the context of Cameroon.^r^ In the context of a campaign-based VAS distribution in Senegal, Horton et al.^41^ reported a cost per child covered of ∼1.25 USD, excluding vitamin A capsules and deworming tablets.^s^ In this setting, the largest cost burden in that context (42.9%) was incurred at the regional level, with 27.4% at the district level, and 29.7% at the local level. Per diems for supervision and incentive payments represented the largest budget share (48.1%), with personnel time costs being the next largest share (34.5%), 8.0% of the total budget was dedicated to training, with the residual allocated to transportation, communications, and other unspecified uses. GiveWell^44^ currently reports that VAS can be delivered for ∼1.00 USD per child reached.

The results reported here, which demonstrate clear differences in the costs and coverage, especially in rural versus urban areas, can suggest which costs might be trimmed without compromising coverage, and what additional expenses might be needed to increase coverage in low-coverage areas.^t^ For example, the proportion of monthly administrative expenses in total program costs was much higher in Yako than in Kombissiri, suggesting that trimming these expenses in Yako could generate improvements in cost-effectiveness. To take another example, VAS coverage in urban areas was consistently lower than that in rural areas, especially for very young children (6 to 11 months of age). Cost data reveal that much less was spent in urban areas than in rural areas, and that spending in urban areas on CWG transportation/communication and community mobilization were especially low. Hence, increased spending in urban areas on these important cost categories may increase coverage and also improve cost-effectiveness. Lastly, but not exhaustively, the data identified concerns regarding JVA+ program sustainability, especially regarding reliance on national and international stakeholders to cover CHW and capsule costs, and the high (time) costs paid by some caregivers in rural areas.

This study had several limitations. First, data were only collected on VAS costs and coverage associated the JVA+ event. Some VAS may have been provided through health clinics, especially for the youngest group of the target population (6 to 11 months of age) and caregivers responding to PEC surveys might have reported JVA+ provision for these children who instead received low-dose vitamin A capsules during scheduled vaccination visits.^45^ However, given that Burkina Faso did not have a clinic-based VAS program in place at the time of this study, we expect there to be very few of these cases. Second, this study focused on a single JVA+ event; results may have been different if multiple JVA+ events had been studied. That said, we do not believe that the 2021/2022 event was in any way different from previous or subsequent events in these health districts. Third, the 2 health districts chosen for this study were not randomly selected from all health districts in Burkina Faso. Instead, they were chosen based on (in this order) security, accessibility, and the presence of existing partnerships; all of these factors may have contributed to different JVA+ activities and costs, and also to higher-performing JVA+ events. This raises concerns about external validity of our findings, both within and beyond Burkina Faso. We cannot speak to the possible implications of increased insecurity on the coverage of cost-effectiveness of JVA+ events in Burkina Faso, but we believe our findings, which are consistent with results in the literature from other (secure) settings, can be generalized. That said, if understanding JVA+ coverage and cost-effectiveness on a national scale given wide differences in security and other conditions are important research goals, future studies should adopt other sampling frameworks. Fourth, the analysis does not deal with the issue of children being at risk of consuming vitamin A above the recommended upper limit (UL), which may be a policy concern.^46^ Finally, our analysis depends on several assumptions used to scale costs to health districts and area types. Costs at the national and regional level were scaled *down* to the health district and area type, while costs at the health facility level were scaled *up* to account for unsampled health facilities. The population of children 6 to 59 months of age was used as the scaling-*down* factor, and the number of CSPS clinics was used as the scaling-*up* factor. Other scaling factors could be used and would likely influence specific results, but would unlikely affect the patterns of results.

## Conclusions and General Implications for Policy

In 2018 to 2019, 91% of Burkinabe children suffered from dietary vitamin A inadequacy, even though a vitamin A-fortified edible oils program was in place. Under these circumstances, only VAS programs can meet vitamin A needs and save children's lives. The twice-annual JVA+ program is in place to deliver VAS and other important products and services to children and their caregivers. We found the program to be generally effective, especially for older children within the target population, and for all children residing in rural areas. We found consistently lower VAS coverage in urban areas—particularly among younger children—suggesting that current urban delivery strategies, characterized by shorter campaign durations and some reliance on volunteers, may be insufficient. To improve reach and equity, urban campaigns should be restructured to better reflect the realities of daily wage earners, itinerant workers, and the fragmented nature of health services in informal settlements. Tailored approaches that focus on low-income and slum neighborhoods could help close coverage gaps among the most vulnerable children while improving efficiency. Although overall program costs were high, the prevalence of vitamin A inadequacy was (roughly) uniformly national in scope, so cost-cutting based on regional targeting was not an option. Therefore, improvements in program performance and efficiency are needed to make the JVA+ program fiscally sustainable. Cost burdens across stakeholder groups can undermine fiscal sustainability and may also need to be addressed. In particular, given that caregiver time accounted for up to 20% of total JVA+ event costs in some areas, more attention should be paid to minimizing these hidden burdens. Strategies such as improved CHW scheduling, SMS reminders, and route optimization could help reduce caregiver wait times. Mapping high-delay zones and deploying extra CHWs during peak periods may also enhance efficiency and equity. Acknowledging and addressing these costs could strengthen community trust and improve program sustainability. Tools and methods exist for estimating the costs and cost-effectiveness of programs that deliver VAS; their results can support policy discussions that aim to improve JVA+ and similar programs. Over the longer term, transitioning from JVA+ events to routine delivery of VAS within the healthcare system may be the optimal strategy, and lessons learned from JVA+ events might help in the design of this system.

## Supplemental Material

sj-docx-1-fnb-10.1177_03795721251355015 - Supplemental material Supplemental material, sj-docx-1-fnb-10.1177_03795721251355015

## Notes

a. Technically, the term cost-efficiency is generally used to assess and report the economic efficiency of programs delivering products or services to individuals, but for which no nutritional or health impacts are expected or measured. VAS capsules are designed to deliver nearly the entire amount of vitamin A required to achieve nutritional adequacy, an important nutritional threshold. Therefore, here and throughout, we use the term cost-effectiveness as a measure of VAS program economic efficiency.

b. We use the term “coverage” to identify the number of young children, or the percentage of that population, who received a vitamin A capsule during a given JVA+ event period, regardless of whether or not those covered were vitamin A deficient or had inadequate dietary intake of VA.

c. JVA+ events delivered VAS and deworming tablets to children and screened for acute malnutrition, and also delivered information to caregivers. Our data collection activities and modeling focused only on VAS delivery, which was the driving force behind, and primary funding source for, this and other JVA+ events. Cost calculations exclude inputs and activities not directly related to VAS delivery.

d. Both health districts are located relatively closely to Ouagadougou: Yako is located ∼100 km north and Kombissiri is located ∼40k south of Ouagadougou. Proximity to the Federal Capital made transportation and data collection safer for research teams, our primary concern in selecting field research sites, and logistics easier to manage.

e. In this context, this meant that in each health district 26.3% of enumeration areas sampled were urban and 73.7% were rural.

f. In households with multiple children in the target age range, all received VAS.

g. For maps locating the enumeration areas within health districts, see Figure SM1 (Yako, rural enumeration areas), Figure SM2 (Yako, urban enumeration areas), Figure SM3 (Kombissiri, rural enumeration areas), Figure SM4 (Kombissiri, urban enumeration areas) in the Online Supplementary Materials.

h. Capsules could have been received in the home, at a clinic, or at pre-identified meeting points for VAS distribution. Given the timing of the PEC survey, we expect that most children received VAS via the JVA+ event, although some younger children may have received VAS at local clinics in association scheduled vaccination visits.

i. Over 80% of caregivers surveyed had no formal education, although this number is driven by the large number of caregivers residing in rural areas, where almost 85% of caregivers have no formal education vs 46% in urban areas.

j. Ranges of salaries for specific job titles/categories were obtained from the ministry of health salary + compensation grid; we used the upper bounds of these job title/category-specific salary ranges in our cost calculations.

k. JVA+ event planners aim to cover *all* children in the targeted age bracket (6-59 months of age), and purchase vitamin A capsules, etc. with that goal in mind. However, VAS coverage is never 100%, so the cost/child targeted will be lower than the cost/child reached.

l. For the case of urban areas in the Yako district, Figure SM5 (Online Supplementary Materials) summarizes the methods used to downscale and upscale costs in order to report estimates at the health district level, for rural and urban areas.

m. All costs and cost-effectiveness results are reported in 2022 USD.

n. The selection of cost categories and the specific costs included in each of them were chosen, in part, to assist in the search for JVA+ event design and management changes that could enhance cost-effectiveness.

o. We use JVA+ social mobilization costs as a proxy for the costs faced by local populations.

p. The Yako and Kombissiri heath districts are located in regions that are generally representative of the other regions in Burkina Faso, with the very notable exception of the population-dense Centre (comprised mainly of urban and peri-urban Ouagadougou) and to a lesser degree the population-scarce regions of Sahel, Est, and Cascades. More specifically, the shares of the under-5 population in Yako and Kombissiri are 21.83% and 20.59% (national average, 21.05%), respectively, with population densities (individuals per square kilometer) of 37.2 and 34.7 (national average, 29.8), respectively, and the share of rural population of 88.26% and 90.26% (national average, 77.87%), also respectively. Therefore, since the JVA+ events are similar nationwide and undertaken essentially at the same time, we expect the results generated in this study based on the 2 health districts to be reasonably scalable to much of the rest of the country, again with the possible exception of the Centre region.

q. These authors report cost-effectiveness in terms of 2004 USD.

r. These authors report cost-effectiveness in terms of 2013 USD.

s. These authors report cost-effectiveness in terms of 2016 USD.

t. Field-based observations of JVA+ activities were required confirm these suggested modifications to program design and implementation; the research team gathered and interpreted these observations to support the suggestions presented here.
