# Supplementary material for: The Cost and Cost-Effectiveness of Vitamin A Supplementation: An Assessment of a Vitamin A Days-Plus Event in Burkina Faso
Source: Food Nutr Bull. 2025 Aug 8;46(4):147–63. doi: 10.1177/03795721251355015 (PMC13250235; doi:10.1177/03795721251355015)

**The Cost and Cost-Effectiveness of Vitamin A Supplementation: An Assessment of a Vitamin A Days-Plus Event in Burkina Faso**

**Online Supplementary Material**

The figures below depict the sampling framework used for rural and urban areas within the Yako and Kombissiri health districts. **Figure SM1** shows the overall boundaries of the Yako health district and the clusters used for household selection in rural areas. **Figure SM2** does the same for the the urban area of the Yako health district.

**Figure SM1: Yako Health District and Rural Enumeration Areas**


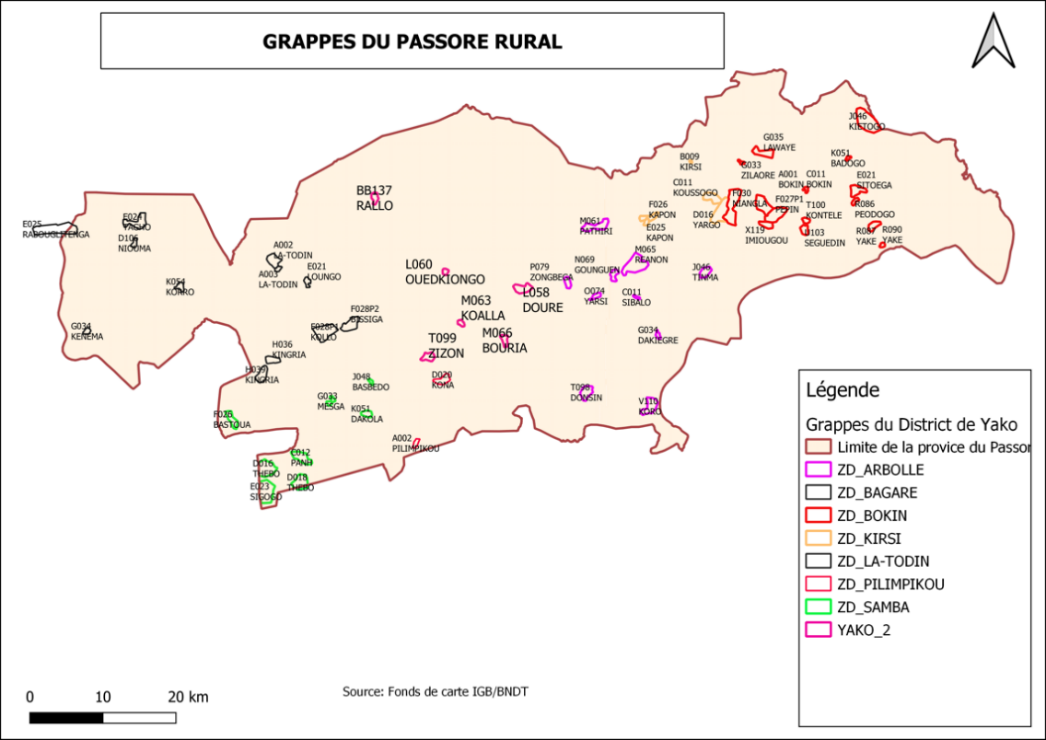


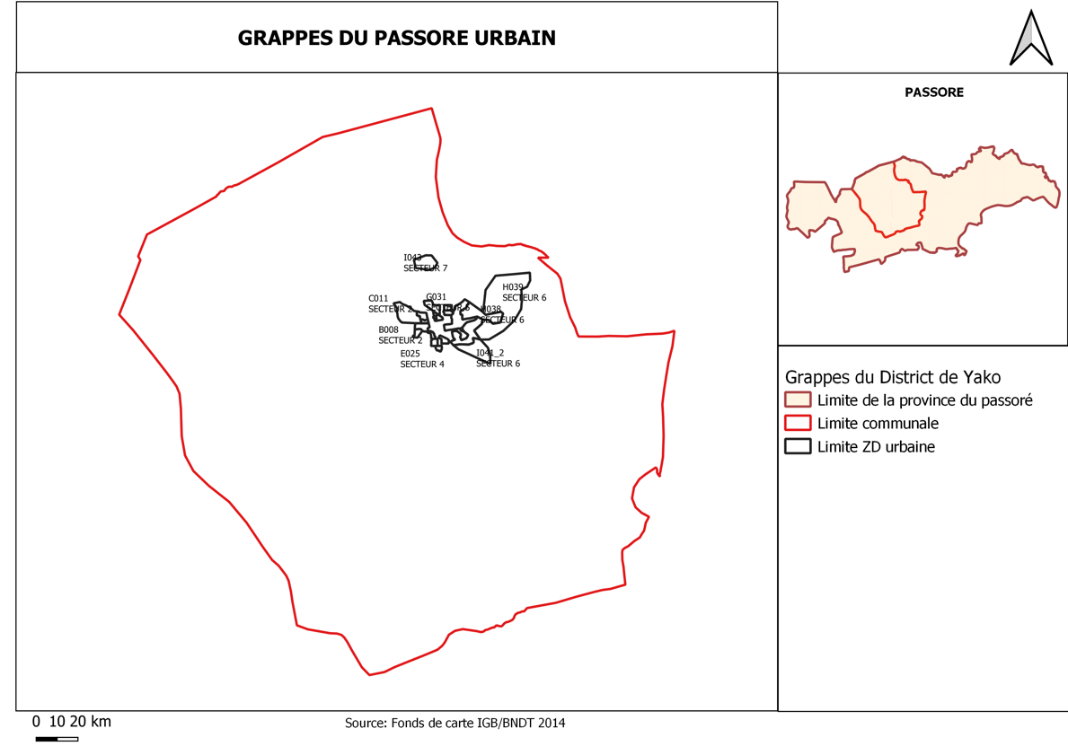
**Figure SM2: Yako District and Urban** **Enumeration Areas**

Similarly, **Figure SM3** and **Figure SM4**, respectively, identify the rural and urban areas of the Kombissiri health district and the clusters used for household selection.

**Figure SM3: Kombissiri Health District and Rural Enumeration Areas**


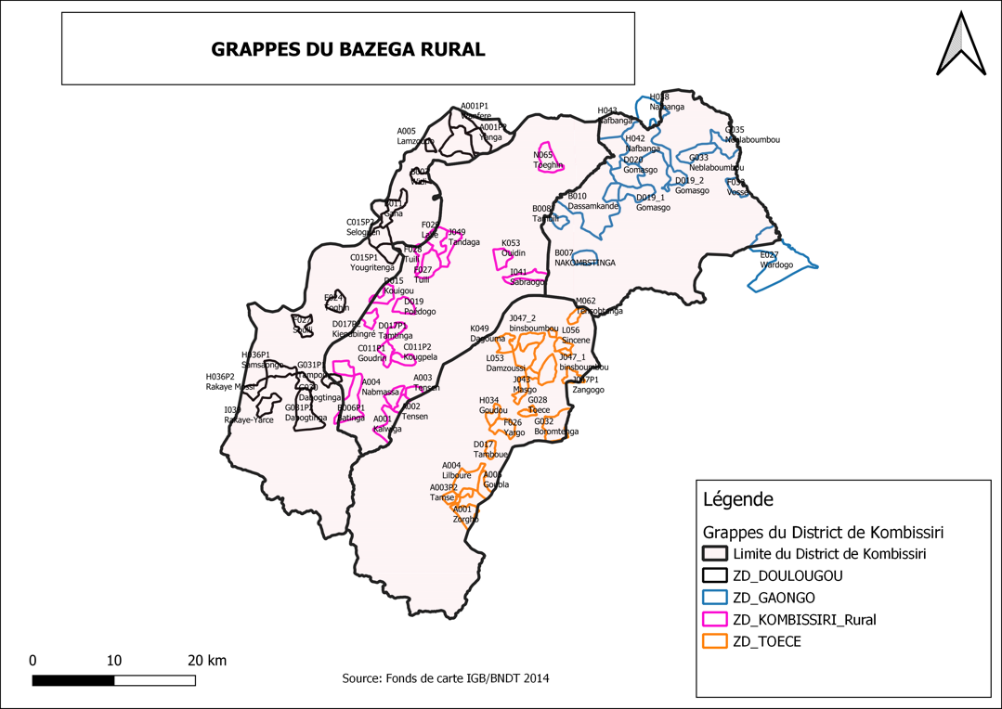


**Figure SM4:** **Kombissiri Health District and Urban Enumeration Areas**


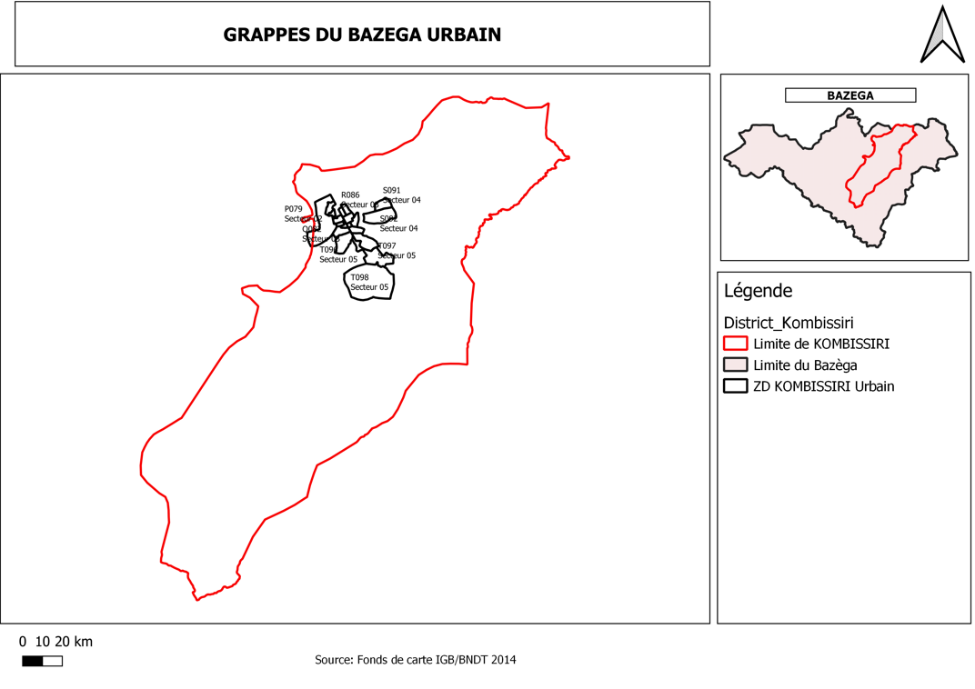


JVA+ activities took place (and hence costs were incurred) at multiple levels, from the household level to the national level. These level-specific costs had to be scaled to arrive at comparable estimates of costs at the health district/area level. **Figure SM5** provides an example of the types of costs involved and the scaling factors used in each case.


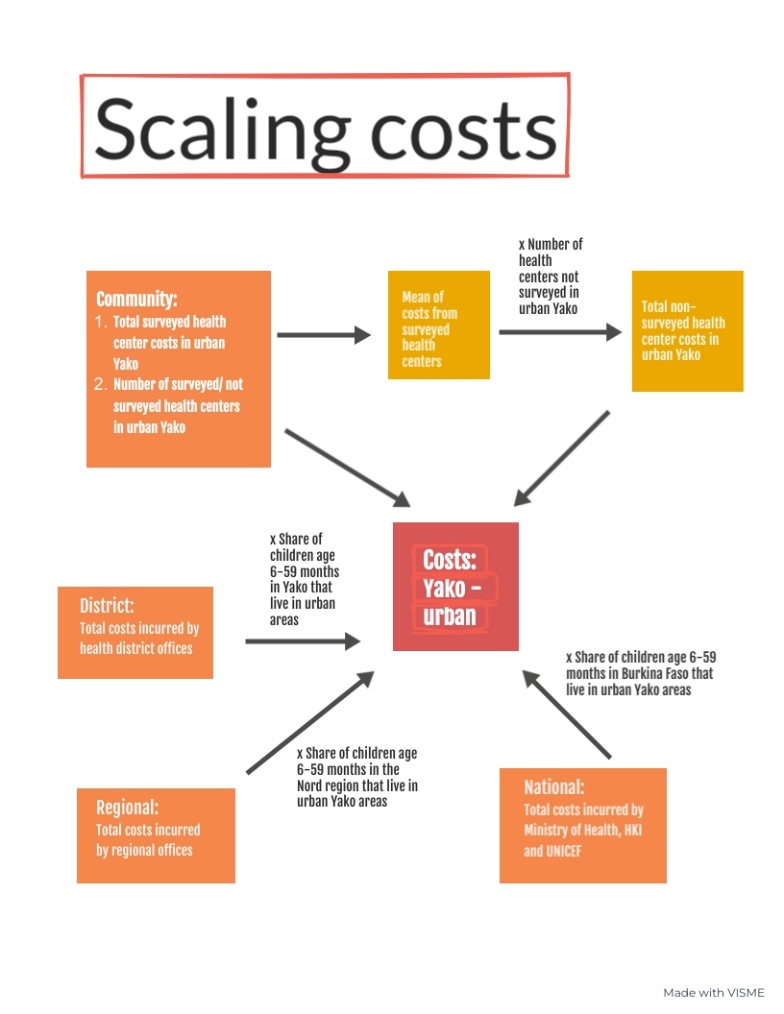
**Figure SM5: Scaling Factors to Address Spatial Differences in Data Sources**

Finally, caregivers invested their time to ensure that their children received VAS during the JVA+ event. **Figure SM6** depicts, by health district and by rural and urban areas, the frequency distributions of the time costs, reported in hours spent waiting for a community health worker (horizontal axis) to deliver the VAS capsule to their children. While the majority of wait times were relatively short, clearly some caregivers dedicated substantial amounts of time to this task, especially in rural areas.

**Figure SM6: Caregiver opportunity costs of time (hours), by Health District, by rural and urban area**


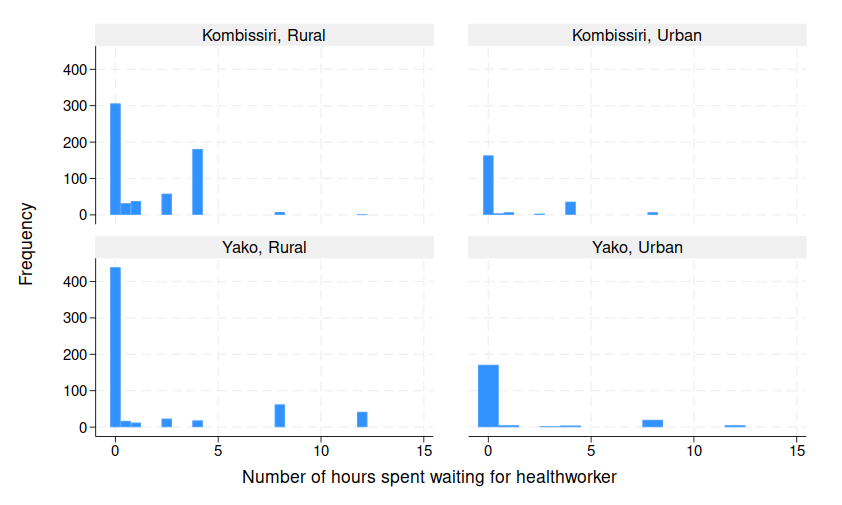

Supplement: sj-docx-1-fnb-10.1177_03795721251355015 - Supplemental material [file sj-docx-1-fnb-10.1177_03795721251355015.docx]
